# Scale and co-management outcomes: assessing the impact of collaborative forest management on community and household resilience in Ghana

**DOI:** 10.1016/j.heliyon.2019.e01125

**Published:** 2019-01-07

**Authors:** Kofi Akamani, Troy Elizabeth Hall

**Affiliations:** a Department of Forestry, Southern Illinois University, 1205 Lincoln Drive, Mail Code 4411, Carbondale, IL 62901, USA; b Forest Ecosystems and Society, College of Forestry, Oregon State University, 321 Richardson Hall, Corvallis, OR 97331, USA

**Keywords:** Economics, Geography, Agriculture, Anthropology, Sociology

## Abstract

Co-management – institutional arrangements that involve the sharing of power, rights and responsibilities between states and resource users – provides a framework for managing common pool resources across multiple scales. However, the scale concept has not received widespread recognition in the assessment of co-management outcomes. This study employed a mixed methods research approach to assess the impacts of collaborative forest management (CFM) on social-ecological resilience at the community and household levels in two forest-dependent communities in the Ashanti region of Ghana. Analysis of qualitative data at the community level indicated that although specific impacts of the CFM program varied within and across the various types of capital assets that shape community resilience, the overall impact of the program on both communities has been positive. At the household level, a statistical comparison of past and current household capital assets showed varying levels of decline in household conditions across the two communities during the implementation of the CFM program. It appears the modest gains from the CFM program at the community level may not have been equitably distributed at the household level. These results suggest that the impact of co-management and other conservation policies may be sensitive to the level at which observation is done. Greater recognition of the importance of scale and cross-scale interactions is needed to inform the formulation of forest policies that contribute to building social-ecological resilience across scales.

## 1 Introduction

Since Hardin's (1968) publication of “The tragedy of the commons,” evidence from decades of research has led to the conclusion that no particular type of institution (states, markets, or communities) can function efficiently, equitably, and sustainably in all common pool resources (Ostrom et al. 1999, 2007; Acheson, 2006; Ostrom, 2007). This realization of the failures in the search for panaceas in the governance of common pool resources provides a strong justification for the co-management concept (Berkes et al., 1989; Acheson, 2013). Co-management refers to an institutional mechanism or a spectrum of institutional mechanisms in which government representatives and resource user-groups, such as local and indigenous communities interact to negotiate formal agreements on the distribution of rights, power, responsibilities and benefits in the resource management process (Yandle, 2003; Plummer and FitzGibbon, 2004; Cinner et al., 2012a; Ayers and Kittinger, 2014; Williams and Tai, 2016). Co-management necessarily takes place at multiple levels of decision-making, as a focus on the community level alone may not always be appropriate in the management of complex common pool resources (Jentoft et al., 1998; Berkes, 2006).

The existing literature highlights several potential benefits in the effective implementation of co-management initiatives, such as enhanced equity, efficiency, and legitimacy in the decision-making process, as well as enhanced community capacity for collective action and conflict management (Jentoft, 2000; Castro and Nielsen, 2001; Carlsson and Berkes, 2005; Plummer and Armitage, 2007). The contributions of co-management to community resilience – the capacity of communities to adapt to drivers of change in ways that contribute positively to their well-being – is also receiving increasing attention (Berkes and Jolly, 2001; Tompkins and Adger, 2004; Akamani and Hall, 2015). Community resilience is often assessed using various types of capital assets that directly shape the well-being of communities and households, as well as their ability to adapt to change (Magis, 2010; Akamani, 2012). Social capital refers to the social relationships that promote cooperative behavior and collective action aimed at addressing common concerns (Fukuyama, 2001; Beckley et al., 2008). Human capital comprises attributes, such as knowledge, skills, and health that influence the ability of actors to make informed decisions and to act upon them to enhance their well-being (Sen, 1997; Flora and Flora, 2013). Economic capital includes financial assets, as well as opportunities for income and employment (Akamani, 2012). Physical capital or built capital refers to the physical infrastructure that facilitates various human activities in communities (Flora and Flora, 2013). Natural capital represents the stock of natural resources upon which communities depend for various ecosystem goods and services (Costanza et al., 1997; Magis, 2010).

In spite of its promise, results from studies on the evaluation of co-management outcomes at various levels of analysis have yielded mixed results of both successes and failures (Gelcich et al., 2006; Fernandez-Gimenez et al., 2008; Cinner et al., 2012b; Mukul et al., 2012; KimDung et al., 2017). The spatial and temporal scales at which co-management evaluation is done could have important implications for the findings (Conley and Moote, 2003). Yet existing studies on common pool resources have generally paid little attention to the scale concept (Berkes, 2008). In this article, we evaluate the resilience impacts of Ghana's collaborative forest management (CFM) program at two levels of analysis: the community and the household. The purpose is to determine whether findings on the impacts of the CFM program at these two levels are equivalent to each other. The broader aim is to understand the importance of scale and cross-scale interactions in the evaluation of outcomes associated with co-management and other conservation policies. In the next section of the paper, we provide an overview of the literature on the scale concept. Next, the context of the CFM program, the geographic region in which the study was conducted, as well as the methods for data collection and analysis will be presented. The following section will then present the results of the study. This will be followed by the discussion and conclusion section.

## 2 Theory

Scale is a critical concept that has received attention in several arenas of resource management (Morse et al., 2009; Termeer et al., 2010; Lebel et al., 2013). Gibson et al. (2000) refer to scale as “the spatial, temporal, quantitative, or analytical dimensions used by scientists to measure and study objects and processes” (p. 219). The scale concept is often used interchangeably with level, which refers to the unit of analysis that is located on the various types of scale (Cash et al., 2006; Berkes, 2008). The choice of spatial and temporal scales plays a crucial role in environmental assessments (O'Brien et al., 2004; Bell and Morse, 2008). From a complex systems perspective, social-ecological systems can be conceptualized as a series of interdependent, semi-autonomous levels with each level exhibiting unique attributes, as well as shared features with the other levels (Holling, 2001; Berkes, 2004). As such, no single level of analysis offers an adequate representation of the entire social-ecological system under study (Berkes, 2004; Akamani et al., 2016).

Cash and Moser (2000) identified three problems that could result from poor attention to multi-level issues in environmental management and assessment exercises. The first is the problem of scale mismatch between the environment and management interventions which the authors refer to as an institutional fit problem. Problems of institutional fit arise when the scale of institutional arrangements for resource management does not correspond with the scale of the social-ecological ecosystems for which they are designed (Folke et al., 2007). For instance, the widespread use of state institutions as solutions for all types of resource management challenges has been criticized as a cause of failure in the management of common pool resources (Berkes, 2004; Ostrom et al., 2007). The second problem is that of scale mismatch between assessment and resource management, i.e. a scale discordance problem. This problem arises from incongruence between the scale of environmental assessment and the scale for which scientific knowledge is required to inform resource management decisions. For instance, much of the existing research on social-ecological systems has focused on large scale patterns and processes, thus failing to offer an adequate understanding of fine scale social processes (Nelson et al., 2007; Akamani, 2012; Berkes and Ross, 2013). Third, there is also the problem of the ignorance of cross-scale dynamics which arises when knowledge on the cause-effect interactions between levels of scale in a complex social-ecological system is missed as a result of a focus of scientific analysis at a single level of scale. For instance, in view of the traditional focus of common pool resources research on the community level of analysis, ignorance of the cross-scale dynamics in the governance of common pool resources remains a critical management challenge that requires the attention of scientists and resource managers (Berkes, 2006, 2008).

Given the inherent problems associated with the traditional focus on a single level of analysis, a multi-level approach has been suggested as a more meaningful way of studying complex social-ecological systems (Cumming et al., 2006; Berkes, 2008). In this regard, Cash and Moser (2000) suggested that an appropriate approach to understanding any given level of analysis is to simultaneously examine neighboring higher and lower levels. In this study, we move beyond the focus on a single level of analysis in co-management assessments by using data at the community and household levels in the evaluation of Ghana's CFM program.

## 3 Methods

### 3.1 Study context

For several decades, forest management in Ghana was guided by the 1948 Forest Policy. In an era of abundant forest resources, this policy focused mainly on the management of forest reserves while off-reserve forests were targeted for the exploitation of timber without replacement (Kotey et al., 1998; Koranteng, 2000). Over the years, rapid degradation of off-reserve forests ensued due to the absence of an effective management mechanism (Koranteng, 2000). At the same time, the growing rural population and declining forest resources led to widespread encroachment on forest reserves. The Forest and Wildlife Policy was adopted in 1994 with the broader goal of promoting the sustainable management of all forest resources (within and outside forest reserves) in collaboration with forest-dependent communities and other relevant stakeholders (Kotey et al., 1998; Koranteng, 2000). This new policy created the institutional environment for the implementation of collaborative forest management (CFM) in Ghana (Asare, 2000). According to Nsenkyire (2000), the Forest Services Division of the Ghana Forestry Commission (GFC) defines CFM as “any interaction between the Division and the local people which improves the flow of benefits to local people and enhances forest management” (p. 1). Among the specific projects that have been implemented under the CFM program are the establishment of community-based forest management organizations, and the implementation of agroforestry initiatives for the restoration of degraded forests. Out of all the CFM projects, it is the agroforestry initiatives that have received the most attention.

The origins of modern agroforestry in Ghana could be traced to the introduction of the taungya system by the Government of Ghana in the 1930s as part of a plantation development program (Agyeman et al., 2003; Acheampong et al., 2016). The taungya system originated from Burma in the late nineteenth century as a land management strategy for producing food crops and establishing forest plantations (Akamani and Holzmueller, 2017). The goal for implementing the taungya system in Ghana was to enhance the production of commercial timber and also to address the need for farmlands for food crop production in forest-dependent communities (Agyeman et al., 2003). Under this program, land in degraded forests was allocated to farmers who cultivated their food crops while helping in forest restoration efforts by establishing and maintaining trees. However, the program was terminated in the 1980s due to a number of factors that accounted for its widespread failure, such as lack of equity in benefit sharing, lack of involvement of farmers in decision-making processes, and lack of tree ownership rights by farmers (Mayers and Kotey, 1996; Agyeman et al., 2003; Blay et al., 2008; Kalame et al., 2011).

In 2002 the Modified Taungya System (MTS), which is a modification of the old taungya system, was introduced by the Government of Ghana with support from the World Bank and the Food and Agriculture Organization (Agyeman et al., 2003; Acheampong et al., 2016). In line with the 1994 Forest and Wildlife Policy, the MTS program promotes collaboration and the equitable sharing of benefits and responsibilities among the various stakeholders in the plantation development process (Ros-Tonen et al., 2013; Foli et al., 2017). Under this new scheme, farmers are entitled to all benefits from food crop production during the first four years of plantation establishment until tree canopy closure. Farmers are also entitled to 40% of timber proceeds, the GFC is also entitled to 40%, traditional land owners are entitled to 15% and communities are entitled to 5% (Agyeman et al., 2003; GFC, 2005). While a number of studies have evaluated the outcomes of the agroforestry initiatives (Kalame et al., 2011; Ros-Tonen et al., 2014; Acheampong et al., 2016; Foli et al., 2017), the resilience impact of the CFM program as a whole across multiple scales has not received enough attention.

### 3.2 Community selection

This study was approved by the University of Idaho Institutional Review Board and consent was obtained from all participants. Two forest-dependent communities in the tropical high forest zone of Ghana were purposively selected using theoretical sampling. According to Eisenhardt and Graebner (2007: 27), “theoretical sampling simply means that cases are selected because they are particularly suitable for illuminating and extending relationships and logic among constructs.” Following consultations with staff of the GFC and visits by the research team to several communities, two communities (Kwapanin and Kyirayaso) in the Ashanti region were selected for the study. The selection of the two communities with similar socio-economic characteristics from the same administrative region and the same ecological zone was intended to minimize variations in the drivers of change influencing the two communities besides the implementation of the CFM program.

#### 3.2.1 Kwapanin

Kwapanin is a farming community of 1,301 inhabitants abutting the Afram Headwaters Forest Reserve which covers an area of 201 square kilometers. The Afram Headwaters Forest Reserve was partly destroyed by severe wildfires and massive deforestation in the 1980s. The wildfires also destroyed the community's cocoa farms that served as the backbone of the local economy. Moreover, Kwapanin was involved in the agroforestry project that was suspended in the 1980s. These adverse events had detrimental effects on the socio-economic well-being of the community. Before the CFM program was introduced, about 75% of households in the community depended on the harvesting and sale of food wrapping leaves and other non-timber forest products (NTFPs) from the forest (Mayers and Kotey, 1996).

As part of the introduction of the CFM program, Kwapanin was chosen in 1993 for a pilot project which aimed at enhancing access to NTFPs among residents of forest-dependent communities. Through this project, the GFC assisted Kwapanin in the establishment of an association of leaves collectors among women in the community. Following the establishment of the association, members were granted access to NTFPs in the forest by the GFC in return for assisting the GFC in the protection of the forest. This collaboration between the NTFP collectors and the GFC was widely publicized as a success story in re-establishing good relationships between the GFC and the Kwapanin community.

The renewed ties between Kwapanin and the GFC have been linked to the subsequent involvement of the community in various agroforestry projects. Kwapanin's participation in the agroforestry initiatives started in 2002 with the introduction of the National Plantation Development Program (NPDP). Another agroforestry project that Kwapanin has been involved in is the Community Forest Management Project (CFMP) that was introduced in the community in 2004. Both the NPDP and the CFMP operate under the guidelines of the MTS as described in the previous section of this manuscript. A distinguishing feature of the CFMP is that it is jointly funded by the Government of Ghana and other external organizations, such as the African Development Bank and the Global Environment Facility. As such, participating farmers have the opportunity to interact with external organizations and also to benefit from more funding and logistics support, as well as opportunities to engage in alternative livelihood ventures such as bee keeping and the rearing of sheep. Membership of the CFMP is, however, limited to 50 farmers per community.

#### 3.2.2 Kyirayaso

Kyirayaso is a relatively smaller community with a population of 865 inhabitants which abuts the Tano-Offin Forest Reserve. The Tano-Offin Forest Reserve covers an area of 413.92 square kilometers, of which 178.34 square kilometers has been designated as a Globally Significant Biodiversity Area (Derkyi et al., 2013). In response to threats posed to the forest, Kyirayaso was assisted by Rural Development and Youth Association (RUDEYA), a non-governmental organization, in establishing a Community Forest Committee (CFC) in 2000. The role of the CFC was to serve as a link between the GFC and the community to ensure that local aspirations and ideas are considered in the forest decision-making and management process (Nsenkyire, 2000). Following its establishment, the CFC worked closely with RUDEYA in the execution of various projects, such as forest protection and tree planting. However, the CFC in Kyirayaso declined after the lifespan of RUDEYA's funded project came to an end. Following the demise of the CFC, the GFC assisted Kyirayaso in establishing the Community Biodiversity Advisory Group (CBAG), charged with the responsibility of protecting the section of the Tano-Offin Forest Reserve that has been designated as a Globally Significant Biodiversity Area. The CBAG was still a vibrant organization during the fieldwork for this study, although community members complained about delay in the delivery of support packages from the GFC, such as the provision of equipment and remuneration.

Kyirayaso has also been involved in the MTS agroforestry project since 2004. Similar to the case of Kwapanin, Kyirayaso's involvement in the MTS project was seen as an outcome of the community forest organizations that had earlier been established. Participation in the MTS is open to all community members. However, not all community members are always interested and have the resources and opportunity to be involved in the project.

### 3.3 Qualitative community level assessment

#### 3.3.1 Data collection

A qualitative research approach was employed to gain an in-depth understanding of the outcomes of community participation in the CFM program. Data for the study were generated through key informant interviews and document reviews. Documents were retrieved and reviewed through consultations with relevant governmental and non-governmental organizations, including the GFC and RUDEYA. These documents provided valuable information on the implementation of the CFM program, as well as the key stakeholders involved in the process. Following this, knowledgeable key informants representing the various sectors of the local society in each community (Luloff, 1999) as well as local and external stakeholders in the forest management process were purposively selected using snowball sampling through which the researcher seeks the assistance of research participants in the recruitment of additional participants (Onwuegbuzie and Collins, 2007). Interviews were conducted using interview protocols that contained several open-ended questions on community conditions before, during and after the CFM implementation process. The sampling and interviewing process continued until the point of theoretical saturation, where additional interviews did not yield new insights (Guest et al., 2006). In all, 36 interviews were recorded and transcribed across the two communities at the local, district and regional levels (17 in Kwapanin; 19 in Kyirayaso). A reflexive journal was kept during the interviewing and transcribing process.

#### 3.3.2 Data analysis

An on-going process of data collection and analysis (Ely et al., 1997) was employed in the study. During the fieldwork, interview transcripts were discussed with two researchers who served as peer-debriefers. This on-going process enabled identification of areas that required further probing in subsequent interviews. After the fieldwork was completed, a more systematic data analysis procedure was employed. A deductive analysis process was employed whereby a number of categories and subcategories were generated from the relevant theory at the beginning of the analysis process. A coding manual, containing these categories and their definitions was compiled and tested on five interview transcripts across the two communities separately by the researcher and another coder. Using this multiple coding approach (Barbour, 2001), the list of categories and subcategories, as well as their definitions were revised to reflect a reasonable level of agreement between the two coders. The use of multiple coders was important for reducing potential researcher bias (Pope et al., 2000) that could have emerged from the researcher's familiarity with the region in which the study was conducted.

After developing the coding manual, the coding was done using QSR International's NVivo 9 qualitative data analysis Software. Following the coding process, qualitative comparative analysis (Leech and Onwuegbuzie, 2008) was used to further analyze and interpret the data. This process involved the systematic analysis of differences and similarities between the two cases with regard to the categories and sub-categories. During the process, memoing of emerging theoretical relationships among the categories and sub-categories was done. Also, close attention was paid to deviant or negative cases that contradicted the emerging patterns in the data (Pope et al., 2000). The use of deviant cases “helps refine the analysis until it can explain all or the vast majority of the cases under scrutiny” (Mays and Pope, 2000: 51). Final interpretation of the qualitative data was based on the triangulation of the interview transcripts, field notes and document summaries (Creswell, 2003).

### 3.4 Quantitative household level assessment

#### 3.4.1 Data collection

Using a structured questionnaire, quantitative data on the impacts of the CFM program on household resilience were collected through the administration of a household survey in the two study communities. The process of data collection and analysis followed guidelines in the development of measurement scales (DeVellis, 1991; Spector, 1992) to develop a valid and reliable household resilience assessment instrument. A list of 30 items representing various dimensions of community resilience (natural capital, social capital, economic capital, human capital, and physical capital) was generated based on a comprehensive review of the literature (e.g. DFID, 1999; Wall and Marzall, 2006; Beckley et al., 2008). Items were selected based on their relevance to rural community well-being in Ghana, as well as their relevance to the theoretical linkages between co-management and community resilience. At least four items were selected to represent each of the five capital assets (Akamani and Hall, 2015). Statements were written for each of these items to which participants responded on a 5-point Likert-type scale (1 = strongly disagree, 2 = disagree, 3 = neutral, 4 = agree, 5 = strongly agree). The questionnaire was translated with the help of two research assistants and pilot-tested in two forest-dependent communities close to those selected for the study. The feedback was subsequently used to revise the questionnaire to enhance the clarity of the statements and to ensure consistency in their interpretation. The final questionnaire was then verbally administered to roughly equal numbers of male and female adult representatives of 209 randomly selected households across the two selected communities (104 in Kyirayaso; 105 in Kwapanin). Only households that had been resident in the communities prior to the implementation of the CFM program were selected to participate in the study. During the survey, respondents were asked to rate the current conditions of their households using the list of 30 scale items (current capitals), as well as the conditions of their households prior to the implementation of the CFM program (past capitals). The approach used to capture retrospective data in this study is consistent with other studies on resilience and the evaluation of policy impacts (Wagner and Fernandez-Giminez, 2008; McManus et al., 2012; Yang et al., 2013).

#### 3.4.2 Data analysis

SPSS software version 18 was used to conduct an internal consistency reliability test on the 30-item scale using combined data from the two communities on current capitals. Five scale items with low or negative item-total correlations whose omission would increase the reliability of the overall scale were deleted and the remaining scales were reanalyzed (Spector, 1992). The revised scale, containing 25 scale items, had an acceptable internal consistency reliability (Cronbach's alpha = .71). To explore the factor structure of the resilience scale, exploratory factor analysis (EFA) was conducted on the items using the principal axis factoring method of factor extraction and the oblique (direct oblimin) method of factor rotation (Stevens, 2002; Costello and Osborne, 2005). Following Field (2009), a value of 0.4 was used as the minimum factor loading for assigning items to each of the factors. The analysis resulted in a 13-item scale comprising four factors (Table 1). Internal consistency reliability tests were conducted on the new scale and the results showed that the reliability of each subscale was adequate: bonding social capital (Cronbach's alpha = 0.68); bridging social capital (Cronbach's alpha = 0.77); natural capital (Cronbach's alpha = 0.73); and economic capital (Cronbach's alpha = 0.63).

**Table 1:** Results from factor analysis of current capitals from both communities.

Scale items	Factor loadings			
	Factor 1	Factor 2	Factor 3	Factor 4
My household members trust one another	**.62**	−.01	−.01	.03
My household has effective and visionary leadership	**.59**	.02	−.03	.03
My household members work closely with one another to address household needs	**.59**	−.02	−.12	−.13
My household members are supportive of one another	**.53**	−.04	.14	−.08
My household members have adequate access to wildlife (bush meat) from the forest	−.05	**.89**	−.08	.00
My household members have adequate access to NTFPs from the forest	−.00	**.60**	−.08	.00
My household members have adequate access to timber from the forest	.00	**.60**	.21	−.02
My household members have enough farmland to support viable agriculture	−.05	.04	**.63**	−.04
My household members have adequate access to vibrant markets	−.08	−.11	**.60**	−.01
My household members are gainfully employed	.11	.09	**.57**	.07
My household members work closely with other households to address household needs	−.14	.00	−.08	**−.89**
My household members and other households trust one another	.18	.04	.03	**−.67**
My household members and other households are supportive of one another	.10	−.02	.07	**−.63**
Eigenvalues	2.94	2.06	1.73	1.30
Percentage of variance explained	22.6%	15.8%	13.3%	10.0%
Cronbach's Alpha coefficient	0.68	0.73	0.63	0.77

The bold values represent factor loadings greater than 0.4. These factor loadings were used to determine the variables that were selected as measures of each of the four factors.

Since the derived 13-item scale did not include items on human capital and physical capital, four additional single items from the original scale were included in the derived scale to increase its content validity (nutrition, roads, financial credit, and non-farm sources of income and employment). To further explore changes in household resilience during the implementation of the CFM program, a comparison of past and current household capitals in the two communities was done using paired-samples *t*-test. The results were evaluated at the .05 significance level.

## 4 Results

### 4.1 Impacts of the CFM program on community resilience

This section presents qualitative data on the impacts of the CFM program on the various dimensions of community capitals that constitute sources of community well-being and resilience. Although most of the impacts mentioned by key informants were associated with the implementation of the MTS agroforestry projects, these results are presented within the broader context of related projects that have been implemented under the CFM program as a whole.

#### 4.1.1 Natural capital

Across both communities, key informants' views on the impacts of the CFM program on the condition and access to the communities' forest resources were mixed. First, perceptions on the current conditions of the forest were explored. In both communities, the overwhelming majority of respondents indicated their dissatisfaction with the current conditions of the forest. They cited numerous factors, including illegal logging, wildfires, and lapses in forest management as some of the causal factors behind the degradation of forest resources. Some respondents, however, held the view that the forest is still good because it provides various benefits to community members, such as food and income.

Key informants were also asked about the effectiveness of the various CFM projects in enhancing forest restoration. Responses generally indicated that tree planting projects in both communities have resulted in moderate gains in forest cover on taungya farmlands. In spite of these moderate gains, it was learned that the emphasis on teak plantations is leading to the loss of indigenous tree species. Also, gains in tree planting were reported to have been offset by the high incidence of illegal logging. It was also reported that the agroforestry projects have been less successful in restoring wildlife populations. One respondent in Kyirayaso indicated that the agroforestry initiatives may have contributed to the migration of wildlife species from the forest because of the noise created by the presence of farmers in the forest.

In terms of forest protection, it was learned that both communities have been effective in dealing with wildfires but were less successful in combating illegal logging. Respondents in Kyirayaso also mentioned that participants in the MTS have made some progress by teaming up with the community forest organizations in protecting the forest against fires and illegal logging. Others noted that the implementation of the MTS has reduced the pressure on community members to embark on environmentally destructive practices, such as illegal logging as a source of livelihood. However, Kwapanin has been less successful in dealing with illegal logging due to the lack of well-functioning community forest organizations, as the association of leaves collectors that was formed at the beginning of the CFM program had collapsed.

#### 4.1.2 Social capital

The implications of the CFM program for various dimensions of community social relations, such as community cohesion, trust, and reciprocity were examined. Most respondents mentioned that community participation in the agroforestry projects has promoted common interests among community members.

“Previously, everybody was struggling individually, and now…..you have to form groups. And when you have to form groups, it means, more or less, you're being brought together to pursue a common course.” (GFC staff, Kwapanin)

Related to the common interests, it was also frequently mentioned that the MTS has promoted unity among community members, as participants engage in acts of reciprocity to support one another during the farm preparation process.

“Right now we are united because sometimes when we finish the farm and someone is sick, members of the association can come together to plant. So it's like we're united.” (MTS farmer, Kyirayaso)

One respondent from Kwapanin, however, observed that while unity exists, it only exists at the group level rather than the community as a whole. This is because several farmers' groups have been created in response to the different agroforestry projects that exist on private and public lands in that community. As a consequence, there is bonding social capital among members of each group but weak bridging social capital across the groups.

“As for the unity, it's there but not much. It's on a group basis. It's true that when something happens, we could all come together. But everyone protects their own” (Chainsaw operator, Kwapanin)

Some respondents also mentioned the incidence of conflict among community members resulting from lack of fairness in the management of the agroforestry projects. It was learned that most of the conflicts occur at the initial stages of the land allocation process.

“We frequently fight a lot over boundaries, and that is disturbing……Sometimes when they allot the parcels and one person gets a larger parcel [than others], it also creates problems.” (MTS farmer, Kyirayaso)

It was learned in Kwapanin that poor social relations among some group members had led to a collapse of some of the projects, such as the alternative livelihood component of the CFMP. The main cause was attributed to the lack of willingness of group members to sacrifice to get the work done. Thus, participation in the agroforestry projects in both communities appears to be marked by both conflict and cooperation, although the diversity of groups and projects in Kwapanin suggests higher incidence of conflict than Kyirayaso.

#### 4.1.3 Economic capital

Key informants from both communities reported enhanced access to income and employment opportunities from the CFM program. Respondents mentioned an increase in farm productivity in the communities due to access to fertile farmlands from the forest through the agroforestry projects. The sale of farm produce from the agroforestry initiatives is the major source of income generation for participants in the program.

“As for the taungya, the little I can say is that since it came, it has come to help this community a bit. The help it has helped is that, right now people are able to work. Whoever works hard, I believe when the crops yield, I should say he gets his fair share. Let's say the hardships that were there at the time when the taungya was not here, at this time it has reduced that hardship a bit.” (CBAG member, Kyirayaso)

Besides the sale of farm produce, it was reported that participants in some of the agroforestry projects, such as the CFMP receive payments for some of their farming activities. Also, the MTS serves as a source of employment for non-participants who offer labor during the farm preparation process and the transportation of farm produce.

But overall gains in income have been somewhat marginal. Respondents mentioned the small size of land parcels that are allocated, poor pricing of farm produce as a result of the poor road network, and high cost of farm maintenance among other factors as detracting from the financial benefits of the program.

“As for that, it's not going well. The parcels they allot to us are usually small. So you can usually sell a small quantity, and the rest has to be consumed [by the household].” (MTS farmer, Kyirayaso)

Other respondents also highlighted the seasonal nature of farming and how it combines with the absence of non-farm sources of income and employment to create under-employment in the communities during some parts of the year. In all, the MTS program appears to have had a moderately positive impact on both communities, although both communities appear to be vulnerable due to their overreliance on the MTS as a source of income and employment.

#### 4.1.4 Human capital

Key informants' responses were generally favorable regarding the impacts of the CFM program on the various dimensions of human capital, such as health, knowledge and skills in the two communities. The most frequently mentioned benefit from the agroforestry projects was the abundance of food due to access to fertile farmlands in the forest reserves.

“I think we're comfortable, we get food to eat. Since our cocoa [farms] burned, the farmlands have been laying there. When you grow crops, they don't yield well. But as for the forest, when you cultivate crops, they yield well until four years' time when the trees overshadow the plantain. And every year you farm, they give you another one [farmland]….” (Hunter and elder, Kwapanin)

Although farm produce is available in the communities, some respondents indicated that reduced access to wildlife from the forest and financial difficulties in purchasing meat on the market adversely affect their nutrition. It was also reported that community members' overreliance on the MTS for their food supplies tended to threaten their food security on occasions when the land was not allocated.

“The benefit it has brought us is the food we get, uhuh! Because we didn't farm it last year, we've experienced hardships this year. We don't even get food to eat.” (MTS farmer, Kyirayaso)

Besides nutrition, some respondents also mentioned gains in knowledge and skills about forest management as a result of their involvement in the MTS and other related projects. Community members mentioned gains in knowledge about the importance of the forest, as well as enhanced awareness of their rights and responsibilities associated with forest management. Key informants also mentioned that they have gained skills through various training workshops on nursery establishment, bee keeping and so forth.

Respondents in Kwapanin also mentioned an increase in the size of the local population and an increased availability of farm labor as a result of the influx of migrants in search of jobs and income opportunities created by the agroforestry projects. In sum, both communities appear to have benefitted from improved human capital, although Kwapanin may have gained a bit more than Kyirayaso.

#### 4.1.5 Physical capital

Key informants were also asked about the impacts of the CFM program on the availability and access to various forms of community infrastructure. The results showed few instances of direct provision of infrastructure under the CFM program. Respondents in Kwapanin reported the construction of a road from the community to the taungya sites through the CFMP. The community has also benefited from the construction of a borehole to improve their source of drinking water, although they could not attribute this directly to the CFM program. In Kyirayaso, respondents also reported that local forest organizations negotiated with timber contractors in the area to secure funding for the provision of boreholes and educational facilities.

While the direct provision of infrastructure from the CFM program was low, respondents noted that the implementation of the agroforestry projects has enhanced the ability of participants to provide some facilities for themselves. Respondents in both communities mentioned improvements in the quality and quantity of their housing stock as a result of the MTS.

“The way the community used to be, it's not like that today. Today, it has expanded…..Even if someone goes to cultivate corn, he may go to purchase a plot of land and build two rooms for himself, his wife and children to go and live in it. This has made the community to expand.” (Traditional leader, Kwapanin)

“As for the community, it has changed. Since the taungya started, it has enabled us to get money to build houses.” (MTS farmer, Kyirayaso)

Also frequently mentioned was the enhanced ability of participants in the MTS to access various forms of services outside the community, such as education.

“Most of them are sending their children out of the community. They're sending them to Kumasi [the regional capital], maybe to go and live with someone, get them a good school because they think the community school is not good or they don't have enough teachers.” (Forestry Commission official, Kwapanin)

Given that access to these external facilities are hindered by the poor condition of the roads linking the communities to the larger centers of commerce and services, most respondents felt that the well-being of their communities will improve with the provision of facilities in their communities.

“If they should come and establish some hospital or some clinic here for us, we'll be happy. But there's nothing here. The other day for instance, someone fell sick here. We carried him. There's a river laying here. Immediately we crossed the river, he died on the way and we had to bring him back. If these things are closer to us, I think it will help us.” (NPDP leader, Kwapanin)

In all, Kwapanin appears to have benefitted more from infrastructure development than Kyirayaso, although both communities were generally dissatisfied with the level of community infrastructure, particularly roads, educational facilities, and health services.

### 4.2 Impacts of the CFM program on household resilience

This section presents quantitative data on changes in the various dimensions of household resilience during the implementation of the CFM program based on the results of the *t*-test comparing current household capitals with their past capitals (Table 2). The results showed declines in all capitals in Kwapanin. All the changes were statistically significant (α = .05), with the exception of bonding social capital, as well as income and nutrition levels. The case of Kyirayaso was somewhat mixed. The results showed an increase in bonding social capital, road conditions, and income levels, and a decline in natural capital, economic capital, bridging social capital, and nutrition levels. Only the declines in economic capital and nutrition levels (representing human capital) were statistically significant. There was no change in access to credit during the implementation period of the CFM program.

**Table 2:** Comparison of current and past household capital assets.

Variable	Kwapanin (n = 105)				Kyirayaso (n = 104)			
	Current	Past	*t* value	*p* value	Current	Past	*t* value	*p* value
Bonding social capital	4.65	4.74	−1.47	0.150	4.33	4.26	0.97	0.330
Natural capital	2.29	3.90	−13.10	<0.001^a^	3.30	3.36	−0.49	0.630
Economic capital	3.60	3.89	−2.70	0.008^a^	3.13	3.50	−3.27	0.001^a^
Bridging social capital	3.94	4.18	−2.03	0.045^a^	3.64	3.70	−0.64	0.520
Nutrition	4.31	4.58	−1.75	0.080	3.68	4.06	−2.63	0.010^a^
Roads	1.17	1.55	−3.09	0.003^a^	1.75	1.63	1.14	0.260
Income	2.65	2.78	−0.72	0.470	2.83	2.67	1.07	0.290
Credit	1.57	2.11	−3.55	0.001^a^	1.72	1.72	0.00	1.000

a Indicates significant at p ≤ .05.

## 5 Discussion & conclusion

The assessment of resource management policy outcomes using a single level of analysis is a widespread practice in research on common pool resources (Cash and Moser, 2000; Berkes, 2006; Folke et al., 2007). Similarly, environmental assessments using data from a single point in time is widespread (Bell and Morse, 2008; Cinner et al., 2015). As a result, knowledge on the implications of scale and cross-scale interactions in the implementation of conservation policies, such as co-management remains limited (Cash et al., 2006; Berkes, 2008). In this study, we employed a mixed methods approach in analyzing the impacts of Ghana's collaborative forest management (CFM) program on social-ecological resilience at the community and household levels. Here, we summarize and discuss the key findings.

### 5.1 CFM impacts on community resilience

The results show that community involvement in the CFM program has resulted in variable impacts within and across the different types of capitals that influence community resilience. Human capital appears to have increased in both communities, particularly with regard to access to food, as well as increased knowledge and skills in forest management. While enhanced access to food and reduced incidence of hunger is an important outcome of the MTS project in particular (Kalame et al., 2011), and agroforestry in general (Magcale-Macandog et al., 2010), the long term food security of the communities remains in doubt due to their over-dependence on the MTS project for farmland. Moreover, the CFM program appears to be characterized by the transfer of knowledge and skills from external experts to local communities and this could undermine long term community empowerment and sustenance of traditional ecological knowledge (Foli et al., 2017). While traditional agroforestry systems have the potential to contribute to the long-term sustainability of ecosystems (Bargali et al., 2004, 2009), this potential remains largely under-utilized in the case of Ghana's agroforestry systems.

With regard to social capital, the CFM program was characterized by both conflict and co-operation. While bonding social capital was exhibited among members of farmers' groups participating in the program, conflict also emerged due to increased competition among community members and the perceived lack of fairness in the selection of participants for the MTS project. The potential for capture of benefits by powerful local elite is a well-recognized problem in Ghanaian forest policy (Marfo et al., 2012), as well as the broader co-management literature (Berkes, 2008). The incidence of conflicts in the CFM program suggests that the co-management process has not played the expected role of serving as an arena for the integration of diverse community interests (Jentoft, 2000).

Results on physical capital also showed that key informants in both communities reported an enhanced ability to access higher order services, such as health and educational facilities in urban areas and also an enhanced ability to improve their housing stock through participation in the MTS agroforestry projects. However, most of the essential community infrastructure, such as roads, health and educational facilities within the communities are either non-existent or in poor condition. In addition to having a negative effect on community well-being, the lack of essential community infrastructure could adversely impact the capacity of communities to attract external conservation and development projects (Dulal et al., 2011).

The results also showed that economic capital has been enhanced through income and employment opportunities from community participation in co-managed agroforestry initiatives in the MTS project, a finding consistent with other studies (Ros-Tonen et al., 2013). However, economic benefits are constrained by several factors, including the small size of land allocated to farmers, high cost of farm maintenance, poor pricing of farm produce, and limited opportunities for non-farm sources of income and employment. As Quinion et al. (2010) have noted, the contributions of agroforestry to sustainable livelihoods are often eroded by the adverse impacts of other drivers of change.

With regard to natural capital, key informants from both communities reported an observed increase in forest cover on MTS lands, as well as an increase in access to farmlands through the MTS project. However, other respondents also bemoaned the declining levels of biodiversity resulting from various anthropogenic disturbances, including farming activities associated with the agroforestry initiatives, as well as illegal logging, wildfires, and poor forest protection mechanisms stemming from the dysfunctional state of the community-based forest management organizations. The observed decline in biodiversity in the forest reserves in both communities is consistent with Young's (2006) contention that negotiated agreements, such as those entailed in co-management processes, can sometimes produce negative ecological outcomes.

In all, the community level data show that both communities have had some success in coping and adapting to the CFM program. Community participation in the CFM program, particularly the agroforestry initiatives, as a short term coping strategy has yielded several benefits, notably access to food, as well as income and employment opportunities. These represent major improvements over previous conditions of hunger and poverty prior to the CFM program. Moreover, CFM participants appear to be employing these short term benefits in engaging in long term adaptation strategies, such as investments in their children's education, and the construction of better houses. However, the overall impact of the CFM program has not been transformative and communities still remain vulnerable to future changes in forest policy as well as the effects of other drivers of change.

### 5.2 CFM impacts on household resilience

Results from *t-*tests comparing past and current household capital assets using the quantitative survey data showed that households in Kwapanin had experienced statistically significant declines in natural capital, economic capital, bridging social capital, roads, and access to credit while households in Kyirayaso also experienced significant declines in economic capital and nutrition.

From the results of the factor analysis (Table 1), the three items that loaded on the factor representing natural capital comprise household members' access to wildlife, NTFPS, and timber from the forest reserve. The reported decline in household access to these resources in Kwapanin reflects community level factors, such as the collapse of the leaves collectors' association, and the decline in wildlife populations in the largely degraded Afram Headwaters Forest Reserve. Although natural capital was also reported to have slightly declined in Kyirayaso, this reported change was statistically insignificant and this reflects the relatively pristine state of the Tano-Offin Forest Reserve, as well as the presence of relatively more effective community forest organizations in that community.

The items representing economic capital comprise access to adequate farmland, access to vibrant markets, and gainful employment opportunities for household members (Table 1). The reported decline in household economic capital in both communities is consistent with key informants' complaints about the limited size of land parcels allocated in the agroforestry project, the poor condition of road transportation systems linking the communities to market centers, the seasonal nature of employment in the agroforestry projects, and the absence of viable non-farm sources of income and employment in the communities.

The factor representing bridging social capital was composed of items that represent cooperative relationships, trust, and reciprocity among households within the community (Table 1). The reported decline in bridging social capital in Kwapanin is consistent with the community level findings and may be linked to the increased competition among households for access to benefits from the CFM program, as well as the perceived lack of fairness in the sharing of CFM benefits among households in the community. While these problems were reported in both communities, the presence of several farmers' associations for the various agroforestry projects in Kwapanin increases the likelihood of conflicts among households who belong to different groups.

Households in Kyirayaso also reported statistically significant declines in nutrition levels which could also be attributed to the perceived food insecurity associated with the intermittent cuts in the yearly allocations of farmland for the agroforestry projects. Unlike Kwapanin that was involved in several agroforestry projects, Kyirayaso was only involved in one MTS project, and hence, was more likely to suffer food insecurity from the fluctuations in the project. The reported decline in nutrition could also be attributed to restrictions in the agroforestry projects on the type of food crops that could be cultivated. For instance, Cassava, which is a staple food crop in the Ashanti region, is not permitted on the MTS farmlands and this could be a source of food insecurity among CFM participants who depend on the program for their food requirements (Ros-Tonen et al., 2013; Acheampong et al., 2016).

### 5.3 Synthesis of findings across scales

The results of this study suggest that the impacts of the CFM program at the community and household levels differ from each other. The analysis of the community level qualitative data showed that the CFM program has had variable impacts within and across the various types of capital assets that shape community resilience. However, when comparing current community conditions to conditions before the CFM program, both communities appeared to have experienced modest improvements in their well-being. In contrast with these community level findings, results from the household level quantitative data showed that household well-being in Kwapanin appears to have experienced a substantial decline over the course of implementation of the CFM program. Although patterns of change in household well-being in Kyirayaso appear mixed, they suggest that conditions in that community have also declined marginally.

One plausible explanation for the observed differences between the community and household level findings is that community level assessments hide peculiar vulnerabilities and inequalities at lower levels of scale, such as the household. Much of commons research is based on the glorified notion of the community as a relatively small territory composed of a homogenous group of people with shared interests (Agrawal and Gibson, 1999; Berkes, 2004). However, communities are complex entities (Berkes and Ross, 2016), composed of various levels of social organization, such as the individual, household, neighborhood, village, and ethnic group (Lebel et al., 2008). Failure to recognize the heterogeneity of communities increases the likelihood that the distribution of the costs and benefits of conservation efforts will be inequitable (Brown, 2003; Lebel et al., 2008). As Cash and Moser (2000) have noted, environmental assessments at higher scales where the focus is on aggregate social welfare tend to reveal relatively small costs whereas assessments at lower scales are more likely to reveal the pattern of distribution of costs and benefits.

Another explanation for the observed differences in CFM impacts at the community and household levels is that owing to the complexity of communities and co-management processes (Carlsson and Berkes, 2005; Berkes, 2006), a focus on any single level of observation offers only a partial understanding of co-management impacts as it fails to capture interactions across the different levels of the system. For instance, while household level analysis have revealed that household characteristics, such as access to resources and institutions are important factors shaping households' ability to benefit from co-management of forest resources (Akamani and Hall, 2015), the results of this multi-level analysis show that community level factors, such as the availability of physical infrastructure, effective institutional mechanisms for conflict management, and the structure of the local economy are equally important in shaping the impacts of Ghana's co-managed agroforestry initiatives on household well-being and resilience. These internal community dynamics are also subject to influences from external drivers of change, such as the mechanisms for the implementation of the CFM program (Akamani et al., 2015), national policies toward the development of rural infrastructure, and the effect of macro-economic conditions at the regional, national and global levels. As Pomeroy et al. (2001) have noted, the factors influencing co-management success occur at multiple levels from the individual/household to the community and supra-community levels.

To conclude, our analysis of the impacts of Ghana's CFM program on community and household resilience has revealed that the impact of the program at the community level was moderately positive while its impact at the household level was relatively negative. It appears the modest gains from the CFM program at the community level may not have been equitably distributed at the household level. In all, the multi-level assessment approach employed in this study has provided a more holistic understanding of the dynamic and multi-dimensional nature of community and household capital assets, as well as the cross-scale interactions within and outside the communities that influence these patterns of change in capital assets. Future studies should seek to further explore these dynamic cross-scale relationships by refining the methods and indicators for such multi-level assessments. With regard to policy, the findings highlight the need for co-management initiatives to pay greater attention to the dynamic and complex nature of the community, as well as embrace integrative approaches for addressing cross-sectoral linkages in order to enhance equitable resilience across scales. This calls for a shift in the focus of Ghana's forest policy from co-management to adaptive forest governance that provides flexible, learning-based institutional mechanisms for connecting individuals and organizations across scales in ecosystem-based management of forest resources.

## Declarations

### Author contribution statement

Kofi Akamani: Conceived and designed the experiments; Performed the experiments; Analyzed and interpreted the data; Contributed reagents, materials, analysis tools or data; Wrote the paper.

Troy Hall: Conceived and designed the experiments; Analyzed and interpreted the data; Contributed reagents, materials, analysis tools or data.

### Funding statement

This work was supported by the Department of Conservation Social Sciences (now Department of Natural Resources and Society), University of Idaho, the College of Natural Resources, University of Idaho, and the Graduate and Professional Students' Association, University of Idaho.

### Competing interest statement

The authors declare no conflict of interest.

### Additional information

No additional information is available for this paper.
